# Comprehensive Identification of Key Genes Responsible for Leaf Senescence of Rice (*Oryza sativa* L.) by WGCNA Using Two Independent Aging Datasets

**DOI:** 10.3390/plants14172704

**Published:** 2025-08-30

**Authors:** Xiaoya Zhou, Hua Zhong, Chuntian Yu, Zhaohai Wang

**Affiliations:** 1 Key Laboratory of Crop Physiology, Ecology and Genetic Breeding, Ministry of Education of the P.R. China, Jiangxi Agricultural University, Nanchang 330045, China; 2 Key Laboratory of Agriculture Responding to Climate Change, Jiangxi Agricultural University, Nanchang 330045, China; 3 Cancer Epidemiology Division, Population Sciences in the Pacific Program, University of Hawaii Cancer Center, University of Hawaii at Manoa, Honolulu, HI 96813, USA

**Keywords:** rice, leaf senescence, weighted gene co-expression network analysis (WGCNA), transcriptome

## Abstract

Leaf senescence is the final stage of plant leaf development, closely related to the yield and quality of cereal crops. However, the molecular regulatory mechanism of rice (*Oryza sativa* L.) leaf senescence is not yet very clear. This study conducted weighted gene co-expression network analysis (WGCNA) using two independent senescence-related transcriptome datasets of rice. Modules positively/negatively correlated with leaf senescence were obtained for each dataset. The additional intersection analysis screened out 180 and 248 common genes highly and positively/negatively correlated with leaf senescence. Gene Ontology (GO) and Kyoto Encyclopedia of Genes and Genomes (KEGG) pathway enrichment analyses showed that these identified common genes were mainly enriched in senescence-related biological processes and pathways, such as reactive oxygen metabolism, hormone pathway, cell death regulation, stimulus–response, amino acid metabolism, photosynthetic metabolism, etc. Transcription factors and studied genes were identified from these common genes, finding that transcription regulation, hormone regulation, reactive oxygen species metabolism, and photosynthesis pathways play an essential role in rice leaf senescence. Protein–protein interaction (PPI) network analysis identified 28 key genes probably involved in leaf senescence. Hub network analysis identified 68 hub genes probably participating in leaf senescence. Twelve genes from the PPI network and the hub gene network were selected for RT-qPCR validation of their expression patterns during leaf senescence. The functions of the senescence-correlated genes identified in this study are discussed in detail. These results provide valuable insights into the regulatory mechanisms of leaf senescence in rice and lay a foundation for functional research on candidate senescence genes.

## 1. Introduction

Leaves are the primary photosynthetic organs in plants, responsible for converting light energy into chemical energy and synthesizing organic materials during the growth period. Leaf senescence is the final step of leaf development and is considered to be an evolutionally acquired process that improves nutrient re-utilization and supports plant survival [1]. During leaf senescence, chloroplasts are degraded, and macromolecules, including proteins, lipids, and nucleic acids, are disassembled and transported to growing young organs [2]. Leaf senescence significantly influences crop yield. Premature leaf senescence typically reduces rice yields due to the decline in photosynthetic capacity, whereas delayed senescence can effectively increase the yield [3]. Hence, the precise regulation of leaf senescence is a critical determinant of crop production, making it a key target in plant breeding and biotechnology. Understanding the regulatory network controlling leaf senescence in rice holds significant agricultural importance.

Leaf senescence is governed by a complex molecular regulatory network. With the development of high-throughput sequencing technology, transcriptome analysis has become a powerful tool for uncovering the molecular mechanisms underlying this process. For example, global transcriptome profiling in cotton (*Gossypium hirsutum* L.) during leaf senescence identified 3624 differentially expressed genes, including 519 transcription factors and 960 genes involved in hormone metabolism and signaling [4]. In common wheat (*Triticum aestivum* L.), transcriptome analysis of a premature senescence mutant identified 1012 senescence-induced genes, highlighting the prominent roles of the WRKY family and zinc finger transcription factors in early senescence [5]. Transcriptome analysis of early senescence in the flag leaf of wheat found that the NAC transcription factor and genes associated with the synthesis, transport, and signaling of multiple phytohormones were differentially expressed [6]. Transcriptome analysis of dynamic changes in leaf senescence of tobacco (*Nicotiana tabacum* L.) revealed that carbon metabolism, starch and sucrose metabolism, nitrogen metabolism, and photosynthesis played a crucial role in the regulation of senescence [7]. Comprehensive transcriptome profiling of early/late leaf senescence grafts in potato (*Solanum tuberosum* L.) revealed that senescence-related genes were enriched in chloroplast organization, electron transport chain, and chlorophyll metabolic process [8]. Global transcriptome analysis of alfalfa revealed six key biological processes of senescent leaves and uncovered that the WRKY and NAC families of genes mainly consisted of transcription factors involved in the leaf senescence process [9]. Comprehensive transcriptomic analysis of age-, dark-, and salt-induced senescence revealed that the NAC, WRKY, bHLH, and ARF transcription factor groups were the major senescence-associated transcription factor families that are key regulators of leaf senescence in *Zoysia japonica* Steud. cv Duckchang [10]. Transcriptome analyses of rice (*Oryza sativa* L.) early leaf senescence mutant *ospls1* identified 4827 differentially expressed genes, in which 81 differentially expressed transcription factors, 11 hormone signaling pathway-related genes, some antioxidative, and carbohydrate metabolism-related genes were suggested to play response and regulatory roles in leaf senescence [11]. Comparative transcriptome analysis revealed that genes related to jasmonic acid, ethylene, abscisic acid, and salicylic acid metabolic pathways and transcription factors including the ERF, WRKY, NAC, and bZIP families, regulated leaf senescence in rice under different nitrogen applications [12]. Transcriptome analysis also revealed that phenylpropanoid biosynthesis, photosynthesis, amino acid (AA) transport, and hormone response were the molecular bases for differential aging programs between flag and second leaves during grain-filling in rice [13]. Despite the wealth of transcriptomic data, most prior studies have relied primarily on Gene Ontology (GO) and Kyoto Encyclopedia of Genes and Genomes (KEGG) pathway enrichment analyses of differentially expressed genes. Given that leaf senescence is a complex quantitative trait regulated by multiple genes, network-based approaches may be more effective in uncovering the key pathways and genes involved.

With the development of analytical methods for high-throughput data, weighted gene co-expression network analysis (WGCNA) has emerged as a powerful tool to identify biologically meaningful gene modules and hub genes associated with specific biological processes. WGCNA has been successfully applied in various plant species. In cotton, RNA-Seq and WGCNA revealed key regulatory modules and genes for salt tolerance, identifying 114 transcription factors and 11 hub genes [14]. In wheat, RNA-Seq-based WGCNA revealed the key regulatory module and genes responding to salt stress in roots [15]. In purple sprout of pak choi (*Brassica rapa* L. ssp. chinensis), transcriptome analysis and WGCNA revealed key genes regulating anthocyanin biosynthesis [16]. In tobacco, transcriptome analysis and WGCNA identified amino acid metabolism pathways as key regulators of nitrogen distribution [17]. In soybean (*Glycine max* L.), transcriptome analysis and WGCNA revealed the potential genetic basis of photoperiod-sensitive male sterility [18]. In rice, genome-wide transcriptome analysis and WGCNA revealed that the phenylpropanoid biosynthesis pathway and the hormone biosynthesis pathway played a potential role in triggering programmed cell death of mutant *pir1* [19]. There are a large amount of transcriptomic data in rice; however, many datasets related to leaf senescence have not yet been explored using WGCNA, partly due to earlier limitations in analytical methodologies. As a result, key genes involved in rice leaf senescence remain insufficiently characterized for these datasets.

Relying on a single transcriptome dataset to identify regulatory genes can lead to findings that are context-dependent or specific to particular genotypes or environmental conditions. By integrating multiple transcriptome data on leaf senescence, it is possible to identify core regulatory genes with broader biological relevance. For example, dataset GSE21398 from the GEO database provides expression profiles of flag leaves from both fertile and sterile rice lines during leaf senescence [20,21]; however, it has not been fully utilized to investigate gene regulation in this context. Another dataset (GSE89233) focuses on the molecular bases for differential aging programs between flag and second leaves during the grain-filling stage of rice [13], but the shared molecular mechanisms of senescence between these leaf types have not been systematically analyzed. In this study, we integrated the transcriptomic datasets GSE21398 and GSE89233 to identify conserved regulatory genes involved in rice leaf senescence using WGCNA. By constructing co-expression networks and identifying modules correlated with the senescence process, we screened for genes commonly associated with leaf senescence across both datasets. These shared genes were subjected to functional annotation and regulatory analysis. Our results provide novel insights into the genetic regulation of leaf senescence in rice and contribute to a comprehensive understanding of its molecular network, offering potential targets for future crop improvement efforts.

## 2. Results

### 2.1. Senescence-Correlated Module Screening by WGCNA

**Figure 1:**
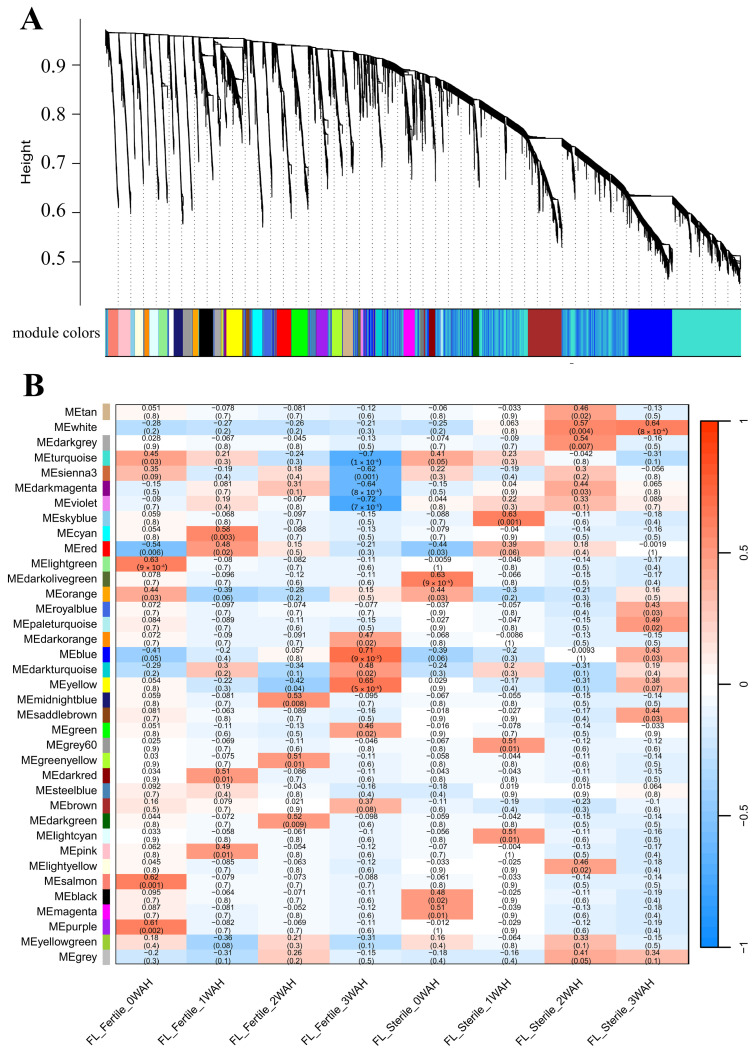
WGCNA module identification. (**A**) Cluster dendrogram and module colors for senescence-related transcriptomic dataset GSE21398. The ordinate “Height” value explicitly refers to the dendrogram branch height in the WGCNA clustering analysis, which reflects the dissimilarity between gene expression profiles. (**B**) Module–sample relationship for senescence-related dataset GSE21398. The numbers in the cell represent module–sample correlations and corresponding *p* values. Each row corresponds to a module, and each column corresponds to a sample at a specific time point. Each cell at the row–column intersection is color-coded by correlation as indicated by the color legend. WGCNA: weighted gene co-expression network analysis.

**Figure 2:**
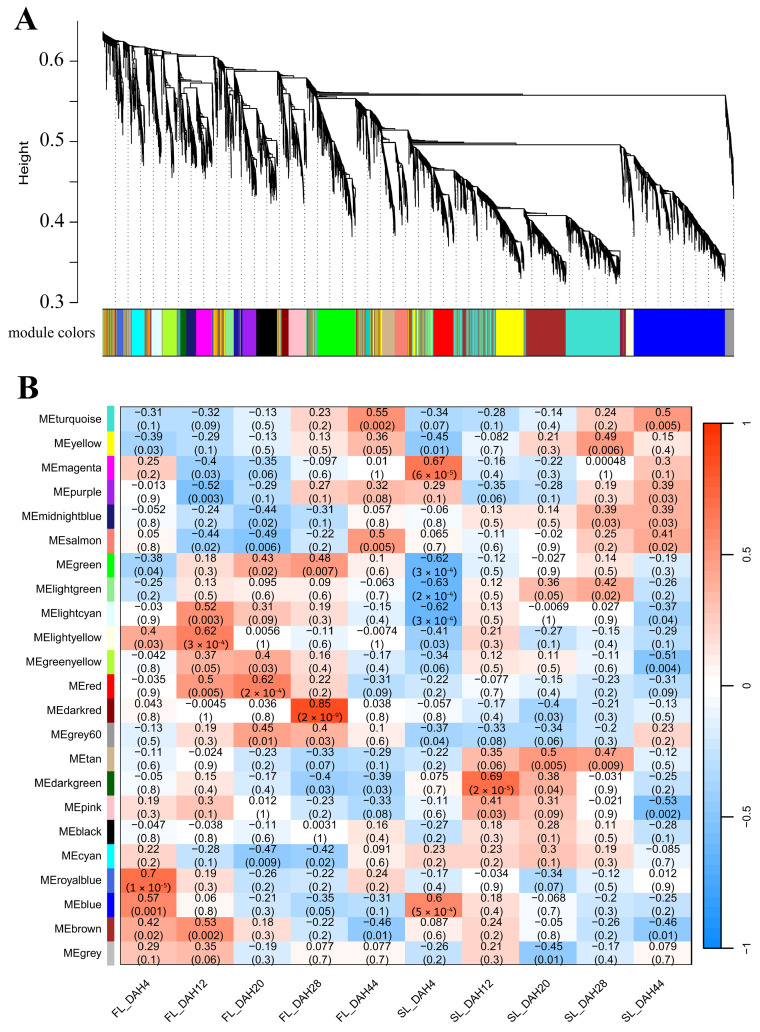
WGCNA module identification. (**A**) Cluster dendrogram and module colors for senescence-related transcriptomic dataset GSE89233. The ordinate “Height” value explicitly refers to the dendrogram branch height in the WGCNA clustering analysis, which reflects the dissimilarity between gene expression profiles. (**B**) Module–sample relationship for senescence-related dataset GSE89233. The numbers in the cell represent module–sample correlations and corresponding *p* values. Each row corresponds to a module, and each column corresponds to a sample at a specific time point. Each cell at the row–column intersection is color-coded by correlation as indicated by the color legend. WGCNA: weighted gene co-expression network analysis.

Genes identified as being consistently involved in senescence across different genetic backgrounds and environmental conditions are expected to have broader biological relevance to rice leaf senescence. To investigate the key genes associated with leaf senescence in rice, two groups of senescence-related transcriptomic datasets, GSE21398 and GSE89233, were screened from the Gene Expression Omnibus (GEO) database, and WGCNA was used to mine biologically meaningful gene modules by framing a gene co-expression network. Samples of GSE21398 contained flag leaves of fertile rice Nipponbare (*Oryza sativa* L. ssp. *japonica*) and its sterile Tos17 insertion mutant lines at four time points (0, 1, 2, and 3 weeks after heading). We selected the top 25% (11,288/45,151) most variable genes in the GSE21398 dataset, and when the soft threshold β = 3, the gene expression matrix conformed to the scale-free with model-fit R^2^ = 0.89. All genes could be divided into 37 modules with respective co-expression similarity (Figure 1A), and the number of genes within the modules ranged from 66 to 1745. Among them, the MEblue module containing 1745 genes showed a persistently positive correlation with leaf senescence both for the fertile and sterile rice groups, while the MEturquoise module containing 1716 genes displayed a continuously negative correlation with senescence (Figure 1B, Table S1). Samples of GSE89233 contained flag leaves and the second leaves of Asian rice cultivar Dongjin (*Oryza sativa* L. ssp. *japonica*) at five time points (4, 12, 20, 28, and 44 days after heading). We selected all 8266 genes in the GSE89233 dataset for WGCNA. Based on the scale-free topology criterion, the soft threshold power of β was set as 26, while the scale-free topology model-fit R^2^ was equal to 0.89. The WGCNA result of GSE89233 could be divided into 23 modules with respective co-expression similarity (Figure 2A), and the number of genes within the modules ranged from 32 to 1724. Of these, the MEturquoise module containing 1715 genes and MEyellow module containing 643 genes showed persistently positive correlations with leaf senescence both for the flag leaf and second leaf groups, while the MEblue module containing 1514 genes and MEbrown module containing 1096 genes displayed continuously negative correlations with senescence (Figure 2B, Table S1).

**Figure 3:**
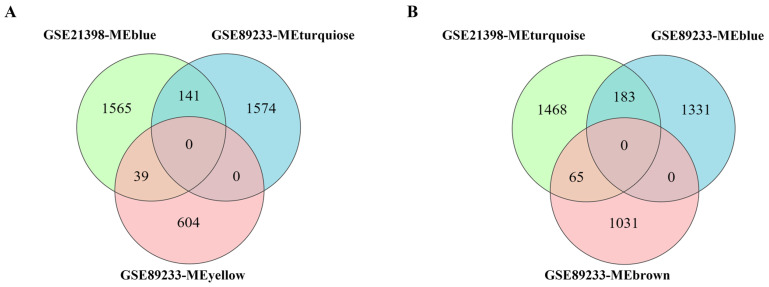
Common and specific senescence-correlated genes between senescence-related datasets GSE21398 and GSE89233. (**A**) Venn diagram of modules positively correlated with senescence. GSE21398-MEblue, module positively correlated with senescence from Figure 1B. GSE89233-MEturquoise and GSE89233-MEyellow, modules positively correlated with senescence from Figure 2B. (**B**) Venn diagram of modules negatively correlated with senescence. GSE21398-MEturquoise, module negatively correlated with senescence from Figure 1B. GSE89233-MEblue and GSE89233-MEbrown, modules negatively correlated with senescence from Figure 2B.

The common senescence-correlated genes screened from multiple rice varieties and multiple groups of experiments can more accurately reflect the universal significance of these genes in the regulation of leaf senescence. In order to further screen the common genes correlated with leaf senescence from both analyzed transcriptomic datasets, we separately integrated the modules positively correlated with senescence (MEblue for GSE21398 and MEturquiose/MEyellow for GSE89233) and modules negatively correlated with senescence (MEturquiose for GSE21398 and MEblue/MEbrown for GSE89233) (Figure 1B and Figure 2B). Eventually, a total of 180 genes were found in the positively correlated intersection, and 248 genes were found in the negatively correlated intersection (Figure 3, Table S2). These commonly identified genes were screened out as highly correlated with leaf senescence and were used for subsequent in-depth analysis.

### 2.2. Enrichment Analysis of Genes Positively/Negatively Correlated with Senescence

**Figure 4:**
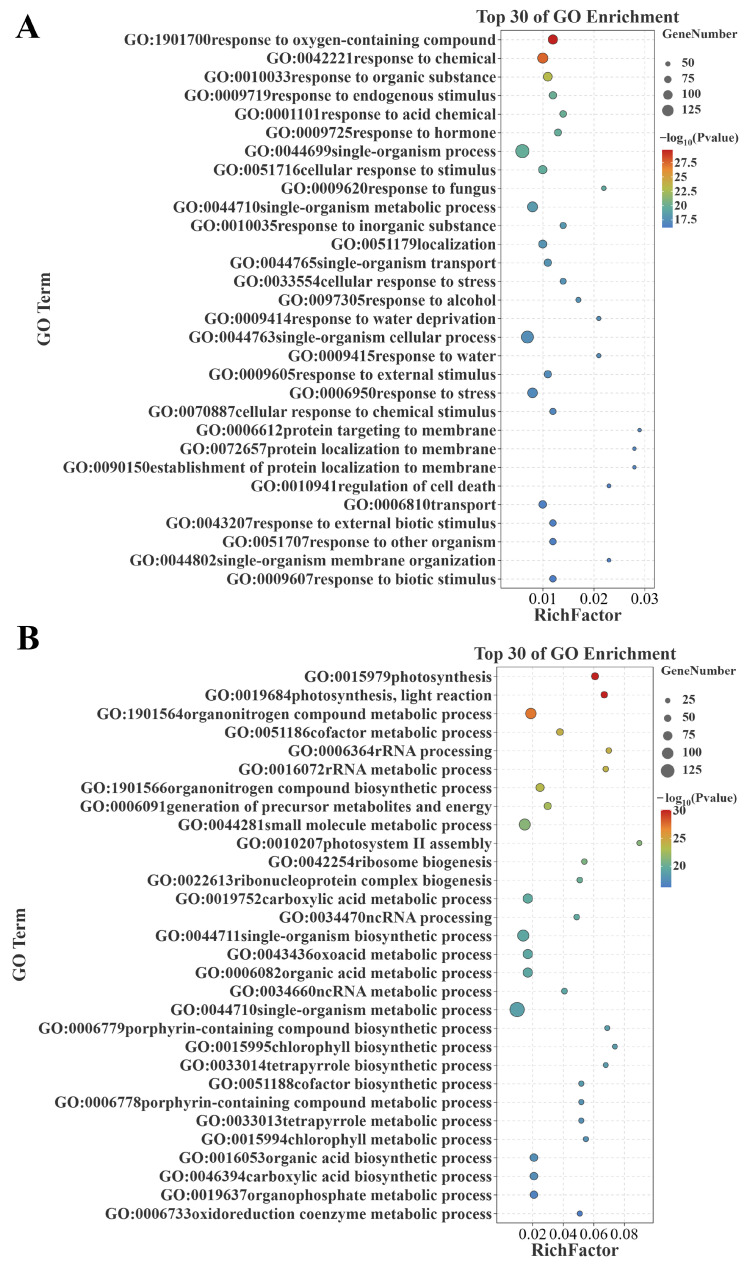
GO subclass (biological process) analysis of differential genes. (**A**) GO biological processes of the commonly identified genes positively correlated with senescence between datasets GSE21398 and GSE89233. (**B**) GO biological processes of the commonly identified genes negatively correlated with senescence between datasets GSE21398 and GSE89233. GO: Gene Ontology.

Functional enrichment analysis was conducted to investigate the potential functions of these genes positively/negatively correlated with senescence. The Gene Ontology (GO) enrichment analysis showed that the genes positively correlated with senescence were assigned to 1000 GO terms (*p* < 0.05), among which, 53, 137, and 810 GO terms were classified under cellular component, molecular function, and biological process, respectively (Table S3). The top 30 GO terms are additionally shown for biological process, and the main terms included ‘response to oxygen-containing compound (GO:1901700)’, ‘response to hormones (GO:0009725)’, ‘regulation of cell death (GO:0010941)’, and ‘responses to various stimuli or stress (GO:0042221, GO:0010033, GO:0009719, GO:0001101, GO:0010035, GO:0097305, GO:0043207, GO:0051707, GO:0009607, GO:0051716, GO:0009620, GO:0033554, GO:0009414, GO:0009415, GO:0009605, GO:0006950, GO:0070887)’ (Figure 4A). Meanwhile, the genes negatively correlated with senescence were assigned to 836 GO terms (*p* < 0.05), among which, 80, 108, and 648 GO terms were classified under cellular component, molecular function, and biological process, respectively (Table S4). The top 30 GO terms are additionally shown for biological process, and the main terms contained ‘photosynthesis related (GO:0015979, GO:0019684, GO:0010207, GO:0006779, GO:0015995, GO:0033014, GO:0006778, GO:0033013, GO:0015994)’ and ‘redox reaction (GO:0043436, GO:0006733)’ (Figure 4B). It is apparent that these genes positively/negatively correlated with senescence are enriched in senescence-associated biological processes.

**Figure 5:**
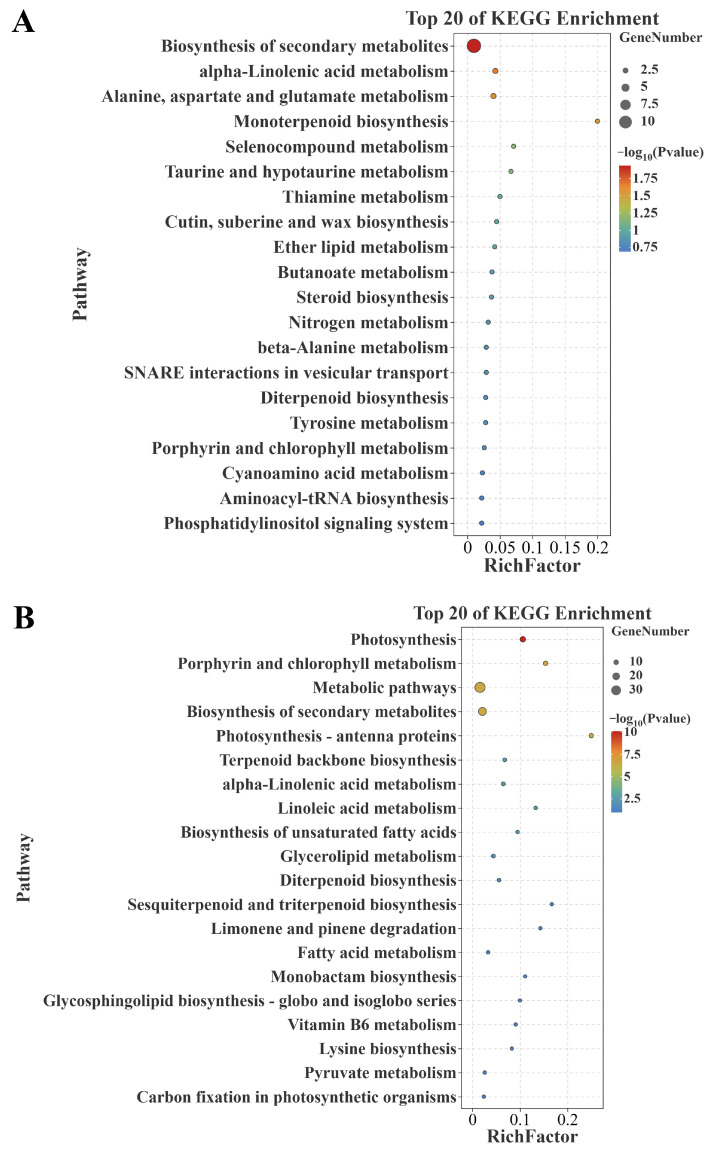
KEGG enrichment analysis of differential genes. (**A**) KEGG pathways of the commonly identified genes positively correlated with senescence between datasets GSE21398 and GSE89233. (**B**) KEGG pathways of the commonly identified genes negatively correlated with senescence between datasets GSE21398 and GSE89233. KEGG: Kyoto Encyclopedia of Genes and Genomes.

The KEGG enrichment analysis displayed the top 20 pathways. The results showed that the genes positively correlated with senescence were significantly enriched in ‘biosynthesis of secondary metabolites (ko01110)’, ‘alpha-linolenic acid metabolism (ko00592)’, and ‘alanine, aspartate, and glutamate metabolism (ko00250)’ (Figure 5A); meanwhile, the genes negatively correlated with senescence were significantly enriched in ‘photosynthesis (osa00195)’, ‘porphyrin and chlorophyll metabolism (osa00860)’, ‘metabolic pathways (ko01100)’, ‘biosynthesis of secondary metabolites (ko01110)’, and ‘photosynthesis (ko00195)’ (Figure 5B). It is apparent that these genes positively/negatively correlated with senescence are enriched in senescence-associated pathways.

### 2.3. Identification of Senescence-Correlated Transcription Factors

**Figure 6:**
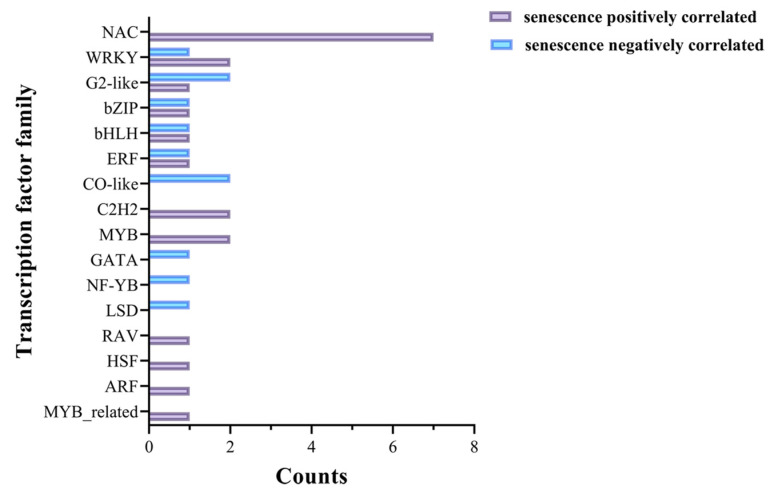
The distribution and number of transcription factor family genes for the commonly identified genes positively/negatively correlated with senescence between datasets GSE21398 and GSE89233.

Transcription factors could play important regulatory roles in the process of leaf senescence [22]. In this study, the screened 180 genes positively correlated with senescence and 248 genes negatively correlated with senescence were submitted to the Plant Transcription Factor Database for transcription factor prediction. In total, 21 positively correlated genes and 11 negatively correlated genes were annotated to encode transcription factors, belonging to the RAV, bHLH, WRKY, NAC, HSF, bZIP, ARF, ERF, G2-like, C2H2, MYB, GATA, NF-YB, CO-like, MYB_related, and LSD transcription factor families (Figure 6, Table S5). These identified transcription factors might play important roles in the leaf senescence process of rice.

### 2.4. Functional Analysis of Identified Senescence-Correlated Genes by Studied Genes

Investigating and displaying known genes provides essential biological context for senescence-related pathways or networks. It will help to integrate our findings with existing knowledge, demonstrating how our newly identified genes might fit into established regulatory pathways of leaf senescence. So far, thousands of rice genes have been cloned and studied for functional research. It is meaningful to investigate whether the senescence-correlated genes identified in this study have been cloned and studied, what their functions are, and whether they are related to leaf senescence. The results showed that among the 428 genes identified to be positively/negatively correlated with senescence, 369 genes were functionally unreported; meanwhile, 59 genes were found to be cloned and studied, among which 20 genes were identified as transcription factors, 14 genes were involved in hormone regulation, 8 genes participated in reactive oxygen species metabolism, 12 genes were related to photosynthesis, and 5 genes participated in other biological processes (Table 1). For transcription factor genes, *Os03g0327800* (*OsNAP*), *Os07g0683200* (*OsNAC103*), *Os03g0782500* (*OsPIL1*), and *Os02g0731700* (*Ghd2*) were reported to play an important role during leaf senescence, while the other 16 genes were found to participate in heading date, photosynthesis, programed cell death, or stress tolerance. For hormone regulation-related genes, 14 genes were reported to be involved in senescence-related signaling pathways including jasmonic acid, salicylic acid, ethylene, abscisic acid, cytokinin, and auxin. For reactive oxygen species metabolism, 12 genes were reported to participate in the clearance, production, or homeostasis of reactive oxygen species. For photosynthesis-related genes, 12 genes were reported to be involved in chlorophyll synthesis, chloroplast development, photosynthesis, and leaf senescence.

Genes that have been clearly reported to be involved in leaf senescence supplied credible evidence for the proposal that the genes identified in this study are involved in leaf senescence in rice. To our knowledge, the other cloned genes have been reported to be involved in biological processes or pathways with a certain connection to leaf senescence, although their senescence phenotype has not been investigated. The participation of these genes in leaf senescence needs further study. The remaining unclonable genes identified in this study are more worthy of further investigation of their functions in leaf senescence. In addition, these studied genes identified from our senescence-correlated genes imply that transcription regulation, hormone regulation, reactive oxygen species metabolism, and photosynthesis pathways play an essential role in rice leaf senescence.

### 2.5. Construction of Protein–Protein Interaction Network of Senescence-Correlated Genes

**Figure 7:**
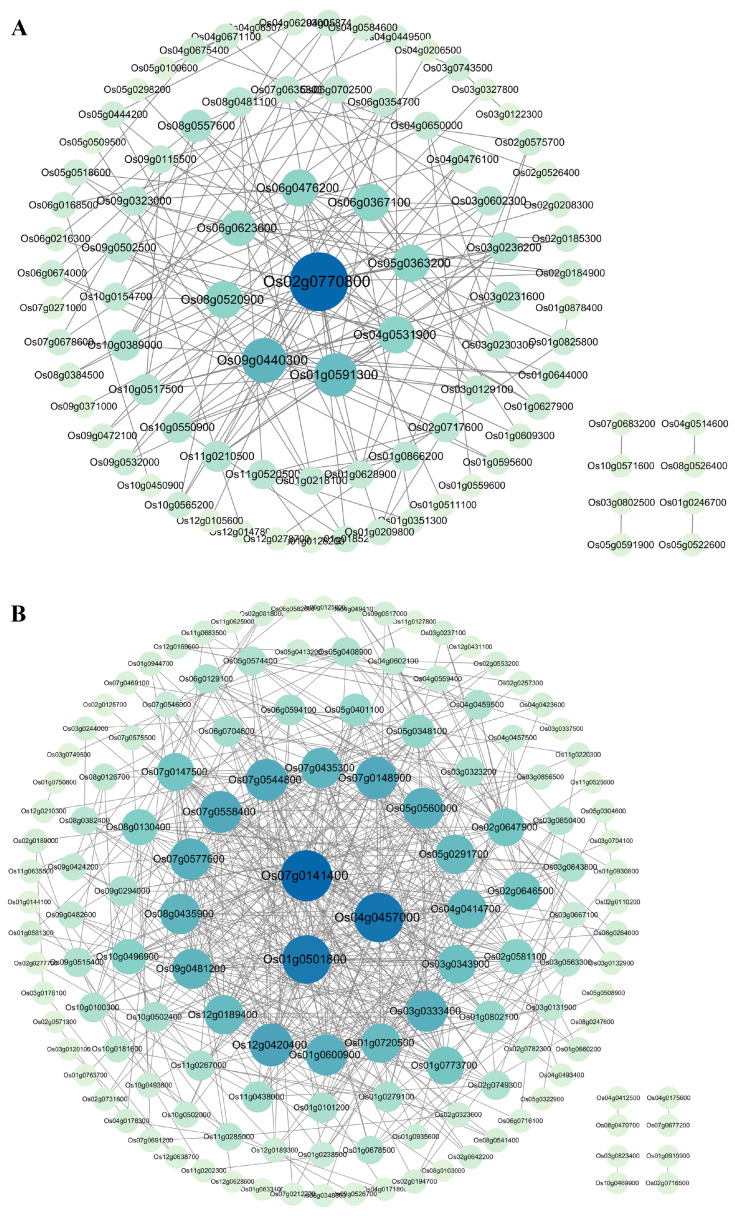
Analysis of the protein–protein interaction (PPI) network. (**A**) The PPI network of the commonly identified genes positively correlated with senescence between datasets GSE21398 and GSE89233. (**B**) The PPI network of the commonly identified genes negatively correlated with senescence between datasets GSE21398 and GSE89233. Different proteins are represented by a node, and their interaction is indicated by the lines. Proteins with more highly connected nodes are indicated by larger circles and bluer colors. Proteins in the middle showed higher interactions.

To understand the functional interaction network of senescence-correlated proteins, the protein–protein interaction (PPI) network was constructed using STRING (https://string-db.org/ (accessed on 17 January 2025)). In the predicted PPI network, highly connected nodes represented the center of the network’s architecture and function. For 180 genes positively correlated with senescence, a total of 89 nodes and 168 edges were involved in the PPI network, in which one gene, *Os02g0770800*, exhibited the highest connectivity degree in the PPI network, followed closely by the eight genes *Os09g0440300*, *Os01g0591300*, *Os04g0531900*, *Os05g0363200*, *Os06g0367100*, *Os06g0476200*, *Os06g0623600*, and *Os08g0520900*, which had relatively high connectivity degrees (Figure 7A). For 248 genes negatively correlated with senescence, a total of 139 nodes and 525 edges were involved in the PPI network, in which three genes, *Os07g0141400*, *Os04g0457000*, and *Os01g0501800*, had the highest connectivity degrees in the PPI network, followed closely by the 16 genes *Os01g0600900*, *Os01g0720500*, *Os03g0333400*, *Os03g0343900*, *Os04g0414700*, *Os05g0291700*, *Os05g0560000*, *Os07g0148900*, *Os07g0435300*, *Os07g0544800*, *Os07g0558400*, *Os07g0577600*, *Os08g0435900*, *Os09g0481200*, *Os12g0189400*, and *Os12g0420400*, which had relatively high connectivity degrees (Figure 7B).

Function annotation revealed that these nine core genes for the positively correlated with senescence PPI network involved various functions, such as nitrate reductase, aldehyde dehydrogenase, glycoside hydrolase, short-chain dehydrogenase/reductase, and starch debranching enzyme, while the 19 core genes for the negatively correlated with senescence PPI network were mainly related to photosynthesis (Table S6). These 28 genes may play important roles in the process of leaf senescence in rice.

### 2.6. Hub Gene Network Analysis

**Figure 8:**
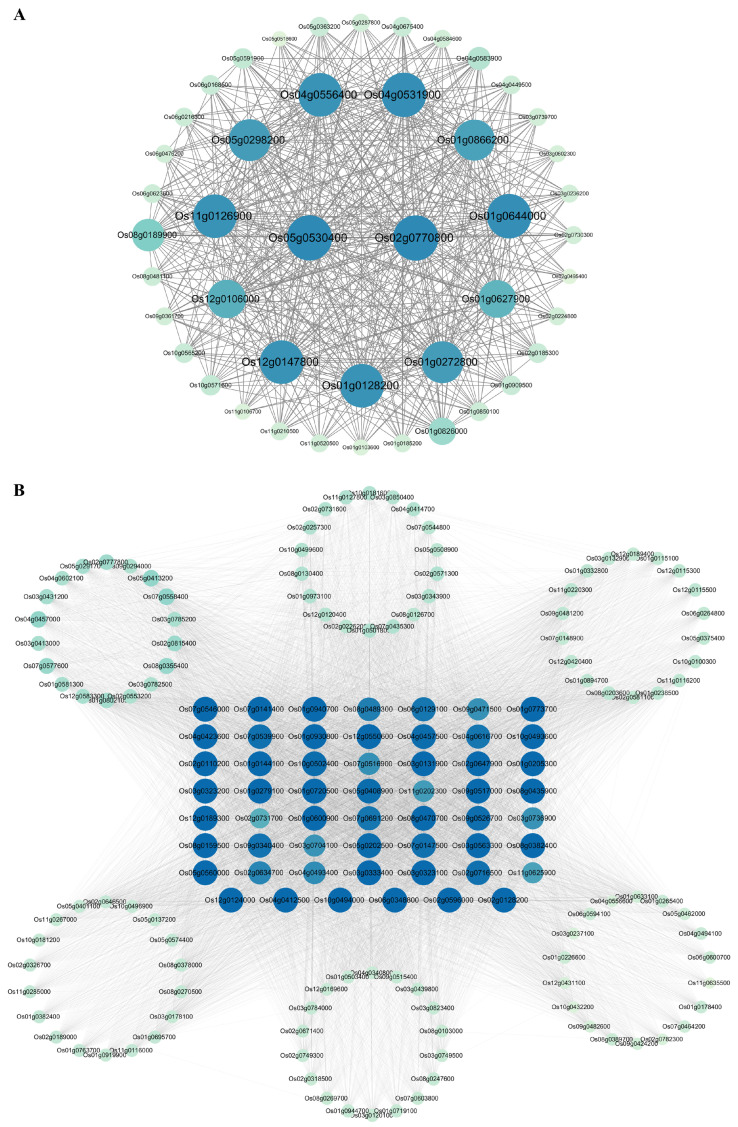
Analysis of hub gene network. (**A**) The hub gene network of the commonly identified genes positively correlated with senescence between datasets GSE21398 and GSE89233. (**B**) The hub gene network of the commonly identified genes negatively correlated with senescence between datasets GSE21398 and GSE89233. Different proteins are represented by a node, and their connection is indicated by the lines. Proteins with higher connectivities are indicated by larger circles and bluer colors. Proteins in the middle showed high connectivity.

Hub genes with high connectivity within significant co-expression modules from the WGCNA are often considered as central regulators within a network. Gene connectivities with a weight value > 0.15 were used for hub gene analysis for the modules positively/negatively correlated with senescence and were visualized using Cytoscape. A total of 45 genes that were positively correlated with senescence constituted the hub network, of which two genes, *Os02g0770800* and *Os05g0530400*, had the highest connectivity degrees, followed closely by 11 genes, including *Os01g0128200*, *Os01g0272800*, *Os01g0627900*, *Os01g0644000*, *Os01g0866200*, *Os04g0531900*, *Os04g0556400*, *Os05g0298200*, *Os11g0126900 Os12g0106000*, and *Os12g0147800*, which had relatively high connectivity degrees (Figure 8A, Table S7). A total of 163 genes that were negatively correlated with senescence constituted the hub network, of which, 55 genes, including *Os02g0128200*, *Os03g0333400*, *Os03g0131900*, *Os09g0517000*, *Os04g0412500*, *Os03g0563300*, *Os01g0930800*, *Os08g0470700*, *Os01g0773700*, *Os08g0435900*, *Os07g0546000*, *Os04g0423600*, *Os07g0141400*, *Os01g0205300*, *Os02g0647900*, *Os01g0600900*, *Os10g0494000*, *Os12g0550600*, *Os01g0279100*, *Os08g0382400*, *Os02g0596000*, *Os01g0720500*, *Os03g0323100*, *Os03g0323200*, *Os06g0348800*, *Os10g0493600*, *Os12g0189300*, *Os05g0560000*, *Os12g0124000*, *Os02g0110200*, *Os01g0940700*, *Os08g0159500*, *Os01g0144100*, *Os05g0408900*, *Os04g0457500*, *Os10g0502400*, *Os05g0202500*, *Os09g0526700*, *Os07g0539900*, *Os07g0147500*, *Os02g0716500*, *Os09g0340400*, *Os04g0616700*, *Os06g0129100*, *Os07g0691200*, *Os08g0489300*, *Os02g0634700*, *Os04g0493400*, *Os07g0516900*, *Os09g0471500*, *Os03g0704100*, *Os03g0736900*, *Os11g0202300*, *Os11g0625900*, and *Os02g0731700*, had relatively high connectivity degrees (Figure 8B, Table S7).

Function annotation revealed that these 13 hub genes for the positively correlated with senescence hub network involved various functions, such as nitrate reductase, heat shock transcription factor, iron storage protein ferritin 2, NAC transcription factor 10, short-chain dehydrogenase/reductase, glucosyltransferase, etc., while the other 55 hub genes for the negatively correlated with senescence hub network included *CO*-like transcription factor, C2H2 type transcription factor, G2-like transcription factor, hydroperoxide lyase gene, massive photosynthesis-related genes, etc. (Table S8). These 68 identified hub genes may play important roles in the process of leaf senescence in rice.

### 2.7. Validation of Gene Expression by qRT-PCR

**Figure 9:**
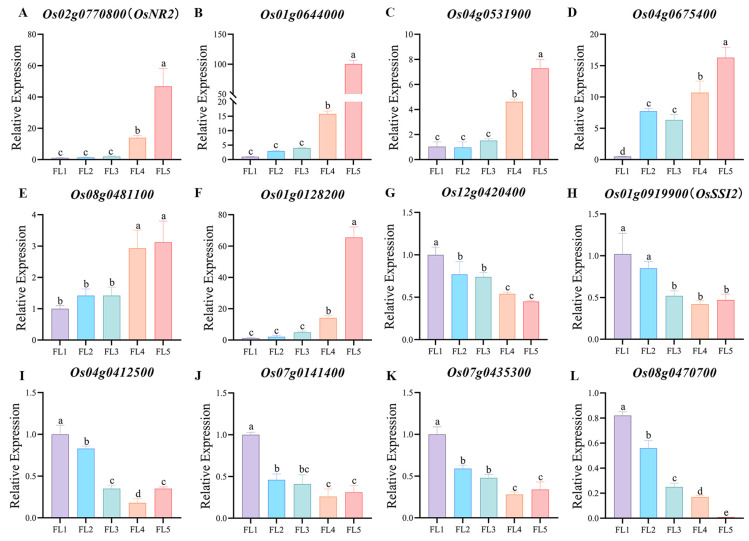
qRT-PCR verification of genes in the PPI network and hub gene network. (**A**–**F**) Expression of the genes positively correlated with senescence. (**G**–**L**) Expression of the genes negatively correlated with senescence. FL1~FL5, flag leaf samples at five distinct stages during leaf development to senescence (FL1, booting stage; FL2, flowering stage; FL3, milk-ripe stage; FL4, waxy-ripe stage; and FL5, fully-ripe stage). Data represent the mean ± SD of three biological replicates. Different letters indicate that values are statistically different based on a one-way ANOVA.

To verify the reliability of the results, six genes positively correlated with senescence and six genes negatively correlated with senescence were selected from the PPI network and hub gene network for qRT-PCR analysis by using flag leaf samples of 93-11 (*Oryza sativa* L. ssp. *indica*). Expectedly, during the process of leaf development to senescence, the six genes *Os02g0770800* (*OsNR2*), *Os01g0644000*, *Os04g0531900*, *Os04g0675400*, *Os08g0481100*, and *Os01g0128200* were all up-regulated (Figure 9A–F), while the six genes *Os12g0420400*, *Os01g0919900* (*OsSSI2*), *Os04g0412500*, *Os07g0141400*, *Os07g0435300*, and *Os08g0470700* were all down-regulated (Figure 9G–L). The qRT-PCR results suggested the accuracy and reliability of our analysis. The validation using qRT-PCR on a separate cultivar 93-11 further confirmed the generalizability of our findings beyond a single genetic background. Importantly, *Os02g0770800*, *Os07g0141400*, *Os01g0644000*, *Os04g0531900*, *Os01g0128200*, *Os12g0420400*, *Os04g0412500*, *Os07g0435300*, and *Os08g0470700* were the core genes from the PPI network or hub gene network, which can be prioritized for subsequent transgenic functional validation.

## 3. Discussion

Analyzing a single GEO dataset is often constrained by its specificity to particular individuals or experimental conditions. In contrast, integrative analysis of multiple GEO datasets enables the more accurate and reliable identification of differentially expressed genes with universal relevance to the biological process being studied. The meta-analysis of GEO datasets has been widely applied in human disease research. For example, a study on osteoarthritis and rheumatoid arthritis performed an intersection analysis on three related datasets (GSE1919, GSE82107, and GSE77298), using an independent dataset GSE55235 as a normal control to validate screened differentially expressed genes; this approach established several genes as potential biomarkers for the differential diagnosis of osteoarthritis and rheumatoid arthritis [92]. Similarly, Wu et al. utilized three Crohn’s disease-related datasets (GSE16879, GSE75214, and GSE112366) to identify six lactylation-related hub genes closely associated with the pathogenesis of Crohn’s disease [93].

Building upon the methodological frameworks established by previous investigations, with the aim of eliminating specific errors in the analysis of individual datasets, the present study conducted WGCNA on two independent rice leaf senescence datasets (GSE21398 and GSE89233). Modules that were positively/negatively correlated with senescence for each dataset were obtained, and an intersection analysis was performed to obtain the expression genes that are highly correlated with senescence (Figure 1, Figure 2 and Figure 3). Functional enrichment analysis, protein–protein interaction (PPI) network construction, and hub gene network analysis were subsequently conducted on these intersected genes (Figure 4, Figure 5, Figure 6, Figure 7 and Figure 8). This comprehensive approach allowed us to identify and discuss a large number of regulatory genes that are highly associated with rice leaf senescence.

### 3.1. Transcription Factors Were Identified as Probably Playing Vital Roles in Regulating Leaf Senescence in Rice

Transcription factors are important regulatory elements for gene expression and have been reported to play vital roles in leaf senescence. In this study, a total of 32 genes were identified to be various transcription factors, with the functions of 20 genes having been studied and 12 genes being unknown. Four transcription factor genes, whose roles in the rice aging process have been previously studied, were identified in our analysis (Table 1). Specifically, *OsNAP* (*Os03g0327800*), a NAC transcription factor, was reported to play an essential role in leaf senescence by fine-tuning abscisic acid biosynthesis and directly targeting senescence-associated genes in rice [3]. Another NAC transcription factor, *OsNAC103* (*Os07g0683200*), has been shown to promote leaf senescence upon overexpression, while its mutation delays leaf senescence under both natural and dark-induced conditions [23]. *OsPIL1* (*Os03g0782500*), a bHLH transcription factor, has been reported to function in chlorophyll biosynthesis [24] and leaf senescence [94]. Lastly, *Ghd2* (*Os02g0731700*), a *CO*-like transcription factor gene, integrates environmental signals and aging processes into the developmental process by interacting with different proteins, regulating physiological processes such as the heading stage, drought tolerance, and aging in rice [25].

Another sixteen transcription factor genes were identified in this study whose functions were found to be involved in heading date, photosynthesis, programed cell death, or stress response but were not explicitly involved in aging (Table 1). Specifically, *OsCOL16* (*Os06g0264200*), a *CO*-like transcription factor gene, was found to regulate the heading stage of rice [26]. This gene was also cloned as *OsBBX17* (*Os06g0264200*), regulating saline-alkaline stress tolerance in rice [27]. *OsRAV12* (*Os05g0549800*) functioned in the regulation of heading date in monocotyledonous and dicotyledonous plants, and roles were unveiled for it in the development of rice gynoecium [28]. *OsHHO3* (*Os03g0764600*) interacts with *OsPIL15*, regulating stomatal aperture through abscisic acid signaling [29]. *OsHHO3* (*Os03g0764600*) was also found to regulate the nitrogen utilization efficiency, chlorophyll content, and photosynthetic efficiency of rice [30]. *Os03g0764600* was predicted as a G2-like transcription factor in this study. *OsCGA1* (*Os02g0220400*), encoding a cytokinin-responsive GATA transcription factor, played an important role in chloroplast development [31]. *OsGLK1* (*Os06g0348800*), encoding a G2-like transcription factor, was reported as a key regulator of chloroplast development under the control of light and phytohormones [32]. This gene was also found to play an important role in the regulation of programmed cell death of pollen tapetal cells [33]. *OsLSD1* played a negative role in regulating plant programmed cell death during resistance to blast fungus [34]. *OsLOL1* was found to stimulate programmed cell death of the aleurone layer of rice grains [35]. In this study, *Os08g0159500* was predicted to be a C2H2-type transcription factor and was identified as *OsLSD1* and *OsLOL1*. *OsWRKY45* (*Os05g0322900*), encoding a rice WRKY-type transcription factor, played different roles in biotic or abiotic stress through salicylic acid (SA), jasmonic acid, and abscisic acid signaling [36,37]. *OsWRKY72* (*Os11g0490900*) regulated bacterial blight resistance through the jasmonic acid and abscisic acid pathways [38]. *OsWRKY72* (*Os11g0490900*) also played an important role in mediating reactive oxygen species scavenging to drive heterosis for salt tolerance in hybrid rice [39]. *OsBIERF1* (*Os09g0434500*) is an ethylene-responsive transcription factor, and its expression was found be induced by various biotic or abiotic stresses [40]. *OsNAC15* (*Os07g0684800*) was reported to regulate rice tolerance to cadmium stress and zinc deficiency [41]. Root-specific expression of *OsNAC10* (*Os11g0126900*) improved drought tolerance and grain yield in rice under field drought conditions [42]. *OsEBP89* (*Os03g0182800*), encoding an ERF transcription factor, played a role in the tolerance process of rice to drought stress throughout its entire growth period [43]. The transcription factor *OsNAC17* (*Os03g0327100*) was found to positively regulate several lignin biosynthetic genes and promote lignin accumulation in leaves and roots, eventually contributing to drought tolerance of rice [44]. The NAC transcription factor *OMTN3* (*Os12g0610600*) was reported to negatively regulate drought resistance in rice [45]. This gene was also identified as *OsNAC60* (*Os12g0610600*) regulating rice immunity against the blast fungus *Magnaporthe oryzae* [46]. Overexpression of the MYB-related transcription factor *OsMYBR1* (*Os04g0583900*) conferred improved drought tolerance and decreased ABA sensitivity in rice [47]. Another MYB transcription factor, *JAmyb* (*Os11g0684000*), played a role in the JA-mediated abiotic and biotic stress response in rice [48]. Obviously, the functions of the transcription factors mentioned above have not been clearly involved in leaf senescence, but their studied functions all involve biological processes similar to or correlated with senescence. Due to the pleiotropy of gene functions, there is a high possibility that they can regulate leaf senescence in rice, which needs further study.

To summarize, this study identified transcription factors that may play vital roles in leaf senescence. Four transcription factors with clear functions in the regulation of leaf aging provided certain evidence for the involvement of the genes identified in this study in aging regulation. Meanwhile, the above-mentioned 16 transcription factor genes, which have been cloned and whose functions have been shown to involve heading date, cell death, or stress response, have a high possibility of being involved in leaf senescence regulation; these can be studied as key candidates in the future. Of course, the remaining 12 transcription factors identified in this study, whose functions were totally unknown, deserve to be preferentially investigated for their roles in regulating leaf senescence.

### 3.2. Hormone Pathway-Related Genes Were Identified as Probably Playing Important Roles in Leaf Senescence in Rice

The leaf senescence process of plants usually involves the participation of hormones, including jasmonic acid [95], salicylic acid [95,96], ethylene [96], abscisic acid [97], cytokinin [98], and auxin [99]. This study identified 14 senescence-correlated genes that were reported to mediate hormone pathways (Table 1). Specifically, *OsHPL3* (*Os02g0110200*) was reported to function in defense responses through the jasmonic acid pathway [49], and the *oshpl3* rice lesion mimic mutant shows spontaneous cell death [50]. The expression of *OsATX* (*Os01g0826000*) was induced by jasmonic acid, salicylic acid, abscisic acid, and hydrogen peroxide, probably playing a role in defense or stress responses [51]. *OCP* (*Os04g0650000*) was found to negatively regulate rice blast resistance through mediating jasmonic acid signaling, ethylene signaling, and auxin signaling pathways [52]. *OsOPR1* (*Os06g0216300*) encodes the 12-O-phytodienoic acid reductase involved in jasmonic acid biosynthesis, and the expression level of this gene was induced under jasmonic acid and salicylic acid treatments [53]. In addition, *Os10g0517500* (*RRJ1*) was identified as a jasmonic acid-responsive gene, but its genetic function remains unclear [54]. *OsSSI2* (*Os01g0919900*) was reported to participate in the negative regulation of spontaneous lesion formation and defense response in rice by regulating the expression of salicylic acid response genes [55]. *OsRNS4* (*Os09g0537700*) was identified as a positive regulator of the ABA response in rice seedlings and a negative regulator of light morphogenesis of rice seedlings [56]. *PLDβ1* (*Os10g0524400*) could stimulate the ABA signaling pathway and suppressed the expression of rice *PLDβ1*, which resulted in reduced sensitivity to exogenous ABA [57]. *CYP71D8L* (*Os02g0184900*) was found to participate in rice growth and stress response by mediating the homeostasis of gibberellin and cytokinin, and overexpression of *CYP71D8L* maintained a high chlorophyll content and low reactive oxygen species level in seedlings [58]. *OsMT2b* (*Os05g0111300*) might control the development of rice roots and the germination of seed embryos by negatively regulating the content of cytokinin [59]. Meanwhile, the gene *OsMT2b* (*Os05g0111300*) was found to play a key role in reactive oxygen species production and cell death during the disease resistance process [60]. *OsAAP5* (*Os01g0878400*) positively regulated the expression of the cytokinin oxidase gene *OsCKX2* and might regulate the growth of rice tiller buds by influencing the level of cytokinin [61]. *OsGH3-2*, encoding an indole-3-acetic acid-amido synthetase, was involved in regulating the contents of auxin and abscisic acid in rice, eventually positively regulating the cold tolerance and negatively regulating the drought resistance of plants [62]. *OsGH3-8*, also encoding an indole-3-acetic acid-amido synthetase, played an important role in the growth, development, and disease resistance response of rice regulated by auxin [63]. *Os01g0764800* and *Os07g0592600* were separately identified as *OsGH3-2* and *OsGH3-8* in this study. *OsTIR1* (*Os05g0150500*) mediated auxin signaling transduction and played an essential role in plant growth and endosperm development of rice [64]. Since the above reports lacked attention to the function of these hormone-related genes in leaf senescence, it would be interesting to investigate whether these genes have functions in leaf senescence in the future. In addition, in this study, 38 and 25 hormone-related GO terms were separately enriched for the genes that were positively/negatively correlated with senescence (Table S9). Considering the reported functions of hormones in plant leaf senescence, it is proposed that the hormone pathway-related genes identified in this study probably play important roles in leaf senescence in rice.

### 3.3. Reactive Oxygen Species-Related Genes Were Identified as Probably Playing Important Roles in Leaf Senescence in Rice

It was reported that reactive oxygen species played a vital regulatory function in plant leaf senescence [100]. In the present study, eight senescence-correlated genes were identified whose functions were reported to be related to regulating the levels of reactive oxygen species (Table 1). Specifically, *OsAPX8* (*Os02g0553200*), encoding the ascorbate peroxidase, is a major H_2_O_2_-scavenging enzyme [65]. *WCR1* (*Os01g0921800*) was found to positively regulate the of transcription of the metallothionein gene *MT2b* involved in reactive oxygen species scavenging and thus reduced the accumulation of reactive oxygen species and delayed programmed cell death in the endosperm of rice, eventually reducing grain chalkiness [66]. Thioredoxin *OsTrxm* (*Os12g0188700*) can interact with *OsMESL* to regulate the clearance of reactive oxygen species, playing an important role in regulating the redox process of chloroplast proteins [67]. *DST* (*Os03g0786400*) was reported to regulate the expression of genes related to reactive oxygen species, affect the homeostasis of reactive oxygen species, and regulate the drought tolerance and salt tolerance of rice [68]. *OsSRO1c* (*Os03g0230300*) was found to regulate stomatal closure, drought tolerance, and oxidative stress tolerance by adjusting the content of hydrogen peroxide [69]. *OsProDH* (*Os10g0550900*) negatively regulated the heat tolerance of rice by regulating proline metabolism and reactive oxygen species scavenging [70]. *CDPK13* (*Os04g0584600*) was reported to activate *RBOHH* and promote the production of reactive oxygen species and was crucial for ethylene-induced aerenchyma formation in rice roots under oxygen-deficient conditions [71]. The deficiency of *OsVPE2* (*Os01g0559600*) led to a reduction in the accumulation of substances such as reactive oxygen species and malondialdehyde, increasing the cold resistance of rice [72]. In addition, in this study, 14 and 17 reactive oxygen species-related GO terms were separately enriched for the genes that were positively/negatively correlated with senescence (Table S9). Considering the reported functions of reactive oxygen species in plant leaf senescence, it is proposed that the reactive oxygen species-related genes identified in this study probably play important roles in leaf senescence in rice.

### 3.4. Photosynthesis-Related Genes Were Identified as Probably Playing Important Roles in Regulating Leaf Senescence in Rice

The regulation of photosynthesis is closely related to leaf senescence [101]. Six photosynthesis-related genes were identified in this study, and their functions have been confirmed to be associated with leaf senescence (Table 1). Specifically, *OscpSRP43* was required for chloroplast development and photosynthesis in rice [73]. *PGL3* affected chlorophyll synthesis by regulating the expression levels of genes related to chlorophyll synthesis, simultaneously affecting leaf senescence through the pathway of reactive oxygen species metabolism [74]. *Os03g0131900* was identified as *OscpSRP43* and *PGL3* in this study. *OsPORB* (*Os10g0496900*) encodes the NADPH: protochlorophyllide oxidoreductase, a key enzyme for chlorophyll synthesis; its mutation may cause reactive oxygen accumulation and thus subsequent lesion formation and premature leaf senescence [75]. Geranylgeranyl reductase, which is encoded by the *CHLP* gene, is responsible for phytyl biosynthesis to form chlorophyll, while the light-harvesting-like protein LIL3 is suggested to be required for the stability of Geranylgeranyl reductase; the rice *lil3 chlp* double mutant exhibited lethality at the three-leaf stage, implying the function of *LIL3* and *CHLP* in the regulation of senescence [76]. *Os02g0125700* was identified as *LIL3* in this study. *OsNYC3* (*Os06g0354700*) and *OsSGR* (*Os09g0532000*) are associated with chlorophyll degradation, and mutations in *OsNYC3* and *OsSGR* can both cause the leaves to stay green and delay senescence in rice [77,78]. *DYE1* (*Os08g0435900*) encodes the Lhca4 subunit of light harvesting complex I; impairment of *DYE1* causes high accumulation of chlorophyll and the stay-green delaying senescence phenotype in rice [79]. These reported genes supplied solid evidence for the participation of photosynthesis-related genes in leaf senescence in rice.

This study also identified six cloned photosynthetic genes, but whether they play a role in leaf senescence remains to be studied (Table 1). *OsGluTR* [80], *OsCHLI* [81], *OsChlH* [82], and *OsCRD1* [83,84], all encoding key enzymes in the chlorophyll synthesis pathway, played important roles in chlorophyll biosynthesis and photosynthesis capacity in rice. *Os10g0502400*, *Os03g0563300*, *Os03g0323200*, and *Os01g0279100* were identified as *OsGluTR*, *OsCHLI*, *OsChlH*, and *OsCRD1*, respectively. *GUN4* (*Os11g0267000*) is required for the activation of the ChlH subunit of magnesium chelatase in chlorophyll synthesis [85]. PsbR was known as the 10 kDa Photosystem II polypeptide. *OsPsbR1* (*Os07g0147500*) was found to play key roles in photosynthesis and cold stress response [86]. It would be interesting to investigate whether these six genes have functions in leaf senescence.

Interestingly, 9 and 21 photosynthesis-related GO terms were separately enriched for the genes positively/negatively correlated with senescence (Table S9). Considering that a large number of photosynthesis-related genes have been reported to be involved in leaf senescence as discussed above, it is proposed that the photosynthesis-related genes identified in this study probably play important roles in leaf senescence in rice.

### 3.5. Function of Key Genes in the PPI Network and Hub Gene Network

The key genes in the PPI network and hub gene network are generally considered to be the most important genes in the studied biological processes, which can be used for subsequent gene function verification research. This study identified 28 key genes from the PPI network and 68 hub genes from the hub gene network during the leaf senescence process (Tables S6 and S8). Three key genes have been clearly reported to be involved in rice leaf senescence. Specifically, the photosynthesis-related gene *Os08g0435900* (*DYE1*) [79] is the key gene both in the PPI network and hub network of the negatively correlated with senescence genes. The transcription factor gene *Os02g0731700* (*Ghd2*) [25] is a hub gene that is negatively correlated with senescence. *Os03g0131900* (*OscpSRP43* and *PGL3*) [73,74] was identified as a hub gene that was negatively correlated with senescence. The functions of these genes in leaf senescence have been discussed in detail in the above paragraph, providing evidence for the role of the key candidate genes identified in this study in aging regulation.

In addition, 14 key genes have been cloned to participate in biological processes that may be related to leaf senescence, but their roles in leaf senescence have not been studied due to different research objectives. *OsNR2*, encoding the nitrate reductase, plays an important role in rice yield potential and nitrogen use efficiency [87]. *Os02g0770800* was identified as *OsNR2* in this study as the key gene both in the PPI network and hub network of the positively correlated with senescence genes. *OsALDH7* regulates seed maturation and viability by clearing acetaldehyde produced during lipid peroxidation reactions [88]. *Os09g0440300* was identified as *OsALDH7* in this study as the key gene in the PPI network. *ISA1*, encoding an isoamylase-type debranching enzyme, played an essential role in starch synthesis and endosperm development [89]. *Os08g0520900*, identified as *ISA1* in this study, was the key gene in the positively correlated with senescence PPI network. *OsSPL7* played a critical role in plant growth and balancing reactive oxygen species during biotic and abiotic stress [90]. *Os05g0530400*, which was identified as *OsSPL7* in this study, was a positively correlated with senescence hub gene. *OsFER2*, a ferritin gene, is required for iron- and reactive oxygen species-dependent ferroptotic cell death and defense response against *Magnaporthe oryzae* infection [91]. *Os12g0106000* was identified as *OsFER2* in this study and is a positively correlated with senescence hub gene. In addition, the transcription factor gene *Os11g0126900* (*OsNAC10*) [42] was identified as a hub gene of the positively correlated with senescence hub network. The G2-like transcription factor gene *Os06g0348800* (*OsGLK1*) [33], C2H2 type transcription factor gene *Os08g0159500* (*OsLSD1* and *OsLOL1*) [34,35], and jasmonic acid pathway gene *Os02g0110200* (*OsHPL3*) [49,50] were identified as the hub genes of the negatively correlated with senescence hub network. The photosynthesis-related genes *Os10g0502400* (*OsGluTR*) [80], *Os03g0563300* (*OsCHLI*) [81], *Os03g0323200* (*OsChlH*) [82], *Os01g0279100* (*OsCRD1*) [83,84], and *Os07g0147500* (*OsPsbR1*) [86] were identified as negatively correlated with senescence hub genes. The functions of these genes have been discussed in detail in the above corresponding paragraph. As the key genes or hub genes in the senescence network, their functions in leaf senescence need to be revealed by further exploration.

The key genes that have not been functionally studied will be the focus of future research regarding their functions during leaf senescence (Tables S6 and S8). Homologous gene studies have implied that these genes may play a crucial role in the process of leaf senescence. For example, aldehyde dehydrogenase was found to function in animal cell senescence [102] and plant stress tolerance [103]. The unclonized gene *Os01g0591300* and a studied gene *Os09g0440300* were annotated as aldehyde dehydrogenase and identified as key genes in the positively correlated PPI network; meanwhile, another aldehyde dehydrogenase gene, *Os02g0647900*, was identified as a hub gene in the negatively correlated hub network. UDP-xylose is a donor for protein glycosylation modification [104], and glycosylation modification was found to be closely related to animal cell aging [105]. *Os05g0363200* was identified as a UDP-xylose synthase gene and as the key gene from the positively correlated PPI network. In addition, the glycoside hydrolase gene *Os06g0367100* (positively correlated PPI network), glycosyl hydrolase family gene *Os01g0940700* (positively correlated hub network), glucosyltransferase gene *Os04g0556400* (positively correlated hub network), and UDP-galactose/glucose epimerase gene *Os09g0526700* (positively correlated hub network), all potentially participate in leaf aging regulation through glycosylation modification processes according to their annotation. Short-chain dehydrogenase/reductase was found to be responsive to drought, salt, and cold stresses in *Medicago truncatula* [106]. *Os04g0531900* was identified as a short-chain dehydrogenase/reductase gene and as the key gene from both the positively correlated PPI and hub network. There were a total of 24 key genes in the PPI network and 53 hub genes in the hub network of senescence-correlated genes whose biological functions were not studied; it would be very meaningful to conduct in-depth research on their functions in the process of leaf senescence.

## 4. Materials and Methods

### 4.1. Data Collection

To investigate the regulatory genes involved in leaf senescence, two publicly available gene expression datasets (GSE21398 and GSE89233) were retrieved from the Gene Expression Omnibus (GEO; http://www.ncbi.nlm.nih.gov/geo (accessed on 15 June 2024)). The GSE21398 dataset includes expression profiles generated using the Agilent-015241 Rice Gene Expression 4 × 44 K Microarray platform (Agilent Technologies, Santa Clara, CA, USA) [20,21]. This dataset profiles flag leaves from three independent sterile mutant lines (*pair1*, *pair2*, and *mel1-1*) and their corresponding fertile (homozygous or heterozygous) segregants. Samples were collected at four developmental stages: heading initiation, 1 week after heading (WAH), 2 WAH, and 3 WAH, with three biological replicates per condition. The GSE89233 dataset comprises Illumina HiSeq 2500 mRNA (Illumina, Inc., San Diego, CA, USA) sequencing data from flag leaves and second leaves, sampled at five time points (4, 12, 20, 28, and 44 days after heading) throughout the grain-filling period [13]. Comprehensive details on the experimental design, transcriptome analysis, microarray platforms, and data processing for both datasets have been previously described [13,20,21]. Raw data processing was conducted using R version 4.1.2.

### 4.2. Weighted Gene Co-Expression Network Analysis

WGCNA was performed using the R package ‘WGCNA’ version 1.72-5 [107]. Initially, RNA-Seq data were preprocessed to remove outliers. We selected the top 25% (11,288/45,151) most variable genes in the GSE21398 dataset to construct a weighted gene co-expression network. A soft-thresholding power (β) was determined based on the scale-free topology criterion to construct a weighted adjacency matrix. Co-expression gene modules were then identified using a bottom-up hierarchical clustering approach with the dynamic tree cut algorithm.

To evaluate module similarity and identify biologically relevant modules associated with tissue types, module eigengenes (MEs), defined as the first principal component of each module, were calculated [108]. Modules significantly correlated with tissue types were identified based on a Pearson correlation test with a *p*-value threshold of <0.05. Additionally, the module preservation function within the ‘WGCNA’ package was employed to compute Z-summary statistics, which were used to assess the preservation and robustness of the identified modules across datasets. Modules with a Z-summary score > 10 were considered as preserved. The genes that were not categorized into any gene modules finally were assigned to the grey module.

### 4.3. Gene Functional Enrichment Analysis

Gene Ontology (GO) functional enrichment was analyzed using the topGO online tool of the BMKCloud platform (https://www.biocloud.net/ (accessed on 17 January 2025)). Senescence-correlated genes were mapped to their corresponding GO terms by using the rice genome Oryza_sativa_Japonica.msu_v7.0 as the background gene set.

For Kyoto Encyclopedia of Genes and Genomes (KEGG) functional enrichment, through the use of the Plant GeneSet Enrichment Analysis Toolkit (https://structuralbiology.cau.edu.cn/PlantGSEA/index.php (accessed on 17 January 2025)), the IDs of senescence-correlated genes were converted into UniProtKB AC numbers that can be recognized by the KOBAS database, and then the enrichment analysis plugin of the KOBAS database (http://bioinfo.org/kobas (accessed on 17 January 2025)) was used to obtain the corresponding KEGG enrichment pathways.

### 4.4. Transcription Factor Prediction

Transcription factor prediction was conducted using the Plant Transcription Factor Database (https://planttfdb.gao-lab.org/ (accessed on 10 January 2025)).

### 4.5. Construction of Protein–Protein Interaction Networks

Co-expression network analysis was performed using the String database (https://string-db.org/ (accessed on 17 January 2025)), with the setting of medium confidence (0.4), and the gene interaction network was then optimized using Cytoscape (v3.10.0).

### 4.6. Quantitative Real-Time PCR (qRT-PCR)

Total RNAs were extracted from the leaves of the rice variety “93-11” (*Oryza sativa* L. ssp. *indica*) at five distinct stages during leaf development to senescence (FL1, booting stage; FL2, flowering stage; FL3, milk-ripe stage; FL4, waxy-ripe stage; and FL5, fully-ripe stage), by using TRIZOL reagent (Invitrogen, Carlsbad, CA, USA) following the manufacturer’s instructions. Reverse transcription of the total RNA to cDNA was conducted by using the PrimeScript™ RT reagent Kit with gDNA Eraser (Takara, Beijing, China). Then, qRT-PCR was performed using the Takara TB Green^®^ Premix Ex Taq™ II FAST qPCR (CN830A) kit on the Applied Biosystems QuantStudio 5 (Thermo, Wilmington, DE, USA). The qRT-PCR operation followed our previously published method, using *EF*-1α (*Os03g0177500*, *LOC_Os03g08020*), *ARF* (*Os05g0489600*, *LOC_Os05g41060*), and *Profilin-2* (*Os06g0152100*, *LOC_Os06g05880*) as internal reference genes [109]. Three biological replicates with three technical replicates were used for the qRT-PCR. All primers are shown in (Table S10).

## Figures and Tables

**Table 1:** Functional analysis of identified senescence-correlated genes by studied genes.

Locus Name	Gene Product Name	Gene Symbol	Gene Functional Information	Reference
*Transcription regulation*				
*Os03g0327800*	Plant-specific NAC transcriptional activator	*OsNAP*	Leaf senescence	[3]
*Os07g0683200*	NAC transcription factor	*OsNAC103*	Leaf senescence	[23]
*Os03g0782500*	Phytochrome-interacting bHLH factor-like	*OsPIL1*	Chlorophyll biosynthesis; leaf senescence	[24]
*Os02g0731700*	CONSTANS (CO)-like gene	*Ghd2*	Heading stage; drought tolerance; senescence	[25]
*Os06g0264200*	CONSTANS (CO)-like gene	*OsCOL16; OsBBX17*	Heading stage; saline-alkaline stress tolerance	[26,27]
*Os05g0549800*	RAV transcription factor	*OsRAV12*	Heading date	[28]
*Os03g0764600*	HHO family transcription factor	*OsHHO3*	Stomatal aperture; nitrogen utilization efficiency; chlorophyll content; photosynthetic efficiency	[29,30]
*Os02g0220400*	Cytokinin-responsive gata transcription factor1	*OsCGA1*	Chloroplast development	[31]
*Os06g0348800*	Golden2-like transcription factor	*OsGLK1*	Chloroplast development; programmed cell death	[32,33]
*Os08g0159500*	Rice Zinc finger protein	*OsLSD1; OsLOL1*	Programmed cell death; blast fungus resistance; aleurone layer development	[34,35]
*Os05g0322900*	WRKY transcription factor	*OsWRKY45*	Biotic or abiotic stress	[36,37]
*Os11g0490900*	WRKY transcription factor	*OsWRKY72*	Bacterial blight; salt tolerance	[38,39]
*Os09g0434500*	Benzothiadiazole (BTH)-induced ethylene responsive transcriptional factor	*OsBIERF1*	Biotic or abiotic stress	[40]
*Os07g0684800*	NAC transcription factor	*OsNAC15*	Cadmium stress and zinc deficiency	[41]
*Os11g0126900*	NAC transcription factor	*OsNAC10*	Drought tolerance	[42]
*Os03g0182800*	EREBP transcription factor	*OsEBP89*	Drought stress	[43]
*Os03g0327100*	NAC transcription factor	*OsNAC17*	Drought stress	[44]
*Os12g0610600*	NAC transcription factor	*OMTN3; OsNAC60*	Drought stress; resistance to rice blast	[45,46]
*Os04g0583900*	MYB transcription factor	*OsMYBR1*	Drought stress	[47]
*Os11g0684000*	JA-inducible Myb transcription factor gene	*JAMyb*	Abiotic and biotic stress	[48]
*Hormone regulation*				
*Os02g0110200*	Hydroperoxide lyase gene	*OsHPL3*	Jasmonic acid pathway; spontaneous cell death	[49,50]
*Os01g0826000*	Antioxidant gene	*OsATX*	Induced by jasmonic acid, salicylic acid, abscisic acid; defense or stress responses	[51]
*Os04g0650000*	Oryzain alpha chain precursor	*OCP*	Jasmonic acid signaling, ethylene signaling and auxin signaling pathways; rice blast resistance	[52]
*Os06g0216300*	12-Oxo-phytodienoic acid reductase	*OsOPR1*	Jasmonic acid biosynthesis; jasmonic acid and salicylic acid response	[53]
*Os10g0517500*	Jasmonic acid-responsive gene	*RRJ1*	Jasmonic acid-responsive	[54]
*Os01g0919900*	Fatty-acid desaturase gene	*OsSSI2*	Regulating the expression of salicylic acid response genes; defense response	[55]
*Os09g0537700*	S-Like ribonuclease	*OsRNS4*	ABA response; light morphogenesis	[56]
*Os10g0524400*	Phospholipase D	*OsPLDβ1*	ABA signaling pathway; sensitivity to exogenous ABA	[57]
*Os02g0184900*	Cytochrome P450 monooxygenase	*OsCYP71D8L*	Homeostasis of gibberellin and cytokinin; rice growth; stress response	[58]
*Os05g0111300*	Metallothionein	*OsMT2b*	Negatively regulating cytokinin content; root development; seed germination; cell death; disease resistance	[59,60]
*Os01g0878400*	Amino acid permease 5	*OsAAP5*	Influencing the level of cytokinin; growth of tiller buds	[61]
*Os01g0764800*	Indole-3-acetic acid-amido synthetase gene	*OsGH3-2*	Regulating the contents of auxin and abscisic; cold tolerance; drought resistance	[62]
*Os07g0592600*	Indole-3-acetic acid-amido synthetase gene	*OsGH3-8*	Auxin regulation; plant growth, development, and disease resistance	[63]
*Os05g0150500*	Auxin transport inhibitor response 1	*OsTIR1*	Auxin signaling transduction; plant growth; endosperm development	[64]
*Reactive oxygen species metabolism*				
*Os02g0553200*	Chloroplast ascorbate peroxidase	*OsAPx8*	H_2_O_2_-scavenging	[65]
*Os01g0921800*	White-core rate 1	*WCR1*	Oxygen species-scavenging; programmed cell death; grain chalkiness	[66]
*Os12g0188700*	Thioredoxin	*OsTrxm*	Clearance of reactive oxygen species; redox process	[67]
*Os03g0786400*	Drought and salt tolerance	*DST*	Homeostasis of reactive oxygen species; drought tolerance; salt tolerance	[68]
*Os03g0230300*	Similar to RCD1 gene	*OsSRO1c*	Adjusting the content of hydrogen peroxide; reactive oxygen species scavenging; stomatal closure; drought tolerance; oxidative stress tolerance	[69]
*Os10g0550900*	Proline dehydrogenase	*OsProDH*	Production of reactive oxygen species; heat tolerance	[70]
*Os04g0584600*	Calcium-dependent protein kinase	*OsCDPK13*	Production of reactive oxygen species; formation of root stomata	[71]
*Os01g0559600*	Vacuolar processing enzyme	*OsVPE2*	Reduction in the accumulation of reactive oxygen species; cold resistance	[72]
*Photosynthesis-related*				
*Os03g0131900*	Chloroplast signal recognition particle 43; pale-green leaf	*OscpSRP43; PGL3*	Chloroplast development; chlorophyll synthesis; photosynthesis; leaf senescence	[73,74]
*Os10g0496900*	Protochlorophyllide oxidoreductase B	*OsPORB*	Chlorophyll synthesis; leaf senescence	[75]
*Os02g0125700*	Light-harvesting-like protein	*OsLIL3*	Chlorophyll formation; leaf senescence	[76]
*Os06g0354700*	Chlorophyll degradation gene	*OsNYC3*	Chlorophyll degradation; leaf senescence	[77]
*Os09g0532000*	Stay green gene	*OsSGR*	Chlorophyll degradation; leaf senescence	[78]
*Os08g0435900*	Delayed yellowing1-1	*DYE1*	Chlorophyll accumulation; leaf senescence	[79]
*Os10g0502400*	Glutamyl-tRNA reductase	*OsGluTR*	Chlorophyll biosynthesis; photosynthesis	[80]
*Os03g0563300*	Magnesium-chelatase subunit ChlI	*OsCHLI*	Chlorophyll biosynthesis; photosynthesis	[81]
*Os03g0323200*	Mg-chelatase H subunit	*OsChlH*	Chlorophyll biosynthesis; photosynthesis	[82]
*Os01g0279100*	Catalytic subunit of magnesium-protoporphyrin IX monomethyl ester cyclase	*OsCRD1*	Chlorophyll biosynthesis; photosynthesis	[83,84]
*Os11g0267000*	Genomes uncoupled 4	*GUN4*	Chlorophyll synthesis	[85]
*Os07g0147500*	10 kDa Photosystem II polypeptide	*PsbR1*	Photosynthesis; cold stress	[86]
*Other biological processes*				
*Os02g0770800*	Nitrate reductase gene	*OsNR2*	Nitrogen use efficiency	[87]
*Os09g0440300*	Aldehyde dehydrogenase 7	*ALDH7B7*	Acetaldehyde clearance during lipid peroxidation reactions; seed maturation and viability	[88]
*Os08g0520900*	Isoamylase1	*ISA1*	Starch synthesis; endosperm development	[89]
*Os05g0530400*	Spotted leaf-7	*OsSPL7*	Plant growth and balancing reactive oxygen species; biotic and abiotic stress	[90]
*Os12g0106000*	Ferritin gene	*OsFER2*	Ferroptotic cell death and defense response	[91]

## Data Availability

Data are contained within the article and Supplementary Materials.

## Supplementary Materials

The following supporting information can be downloaded at: https://www.mdpi.com/article/10.3390/plants14172704/s1, Table S1: Gene information of positively/negatively correlation modules; Table S2: Intersection of senescence positively/negatively related genes; Table S3: GO enrichment analysis of genes positively correlated with senescence; Table S4: GO enrichment analysis of genes negatively correlated with senescence; Table S5: Transcription factor information of genes positively/negatively correlated with senescence; Table S6: Function annotation of core/key genes in the PPI network; Table S7: Connectivity relationship of genes positively/negatively correlated with senescence; Table S8: Function annotation of hub genes in the hub network; Table S9: GO terms related to hormones, reactive oxygen species, and photosynthesis; Table S10: All primers used for qRT-PCR analysis.

## Abbreviations

The following abbreviations are used in this manuscript:

WGCNA	Weighted gene co-expression network analysis
GO	Gene Ontology
KEGG	Kyoto Encyclopedia of Genes and Genomes
PPI	Protein–protein interaction
