# Supplementary material for: Comprehensive Identification of Key Genes Responsible for Leaf Senescence of Rice (Oryza sativa L.) by WGCNA Using Two Independent Aging Datasets
Source: Plants (Basel). 2025 Aug 30;14(17):2704. doi: 10.3390/plants14172704 (PMC12430195; doi:10.3390/plants14172704)
Supplement: Supplementary file 1 [file plants-14-02704-s001.zip › Table S6.pdf]

Table S6. Function annotation of core/key genes in the PPI network

Locus name	Gene product name	Gene symbol	Gene functional information	Reference
**Senescence positively correlated genes**				
Os02g0770800	Nitrate reductase	*OsNR2*	Nitrogen use efficiency	Gao et al. 2019
Os09g0440300	Aldehyde dehydrogenase 7	*OsALDH7*	Acetaldehyde clearance during lipid peroxidation reactions; seed maturation and viability	Shin et al. 2009
Os08g0520900	Isoamylase-type debranching enzyme	*ISA1*	Starch synthesis; endosperm development	Utsumi et al. 2011
Os01g0591300	Rice aldehyde dehydrogenase	/	/	/
Os04g0531900	Short-chain dehydrogenase/reductase, putative, expressed	/	/	/
Os05g0363200	UDP-xylose synthase	/	/	/
Os06g0367100	Glycoside hydrolase encoding gene	/	/	/
Os06g0476200	Expressed protein	/	/	/
Os06g0623600	Reductase, putative, expressed	/	/	/
**Senescence negatively correlated genes**				
Os08g0435900	Delayed yellowing1-1	*DYE1*	Leaf senescence	Yamatani et al. 2018
Os07g0141400	23 kDa polypeptide of the oxygen-evolving complex of photosystem II	/	/	/
Os04g0457000	Chlorophyll A-B binding protein, putative, expressed	/	/	/
Os01g0501800	Oxygen-evolving complex	/	/	/

Os01g0600900	Chlorophyll A-B binding protein, putative, expressed	/	/	/
Os01g0720500	Chlorophyll A-B binding protein, putative, expressed	/	/	/
Os03g0333400	Photosystem II 11 kD protein, putative, expressed	/	/	/
Os03g0343900	Ultraviolet-B-repressible protein, putative, expressed	/	/	/
Os04g0414700	Membrane protein, putative, expressed	/	/	/
Os05g0291700	PTAC16, putative, expressed	/	/	/
Os05g0560000	Expressed protein	/	/	/
Os07g0148900	Photosystem I reaction center subunit, chloroplast precursor, putative, expressed	/	/	/
Os07g0435300	Photosystem I reaction center subunit IV A, chloroplast precursor, putative, expressed	/	/	/
Os07g0544800	Oxygen evolving enhancer protein 3 domain containing protein, expressed	/	/	/
Os07g0558400	Chlorophyll A-B binding protein, putative, expressed	/	/	/
Os07g0577600	Chlorophyll A-B binding protein, putative, expressed	/	/	/
Os09g0481200	Photosystem I reaction center subunit, chloroplast precursor, putative, expressed	/	/	/

Os12g0189 400	Photosystem I reaction center subunit N, chloroplast precursor, putative, expressed	/	/	/
Os12g0420 400	Photosystem I reaction center subunit XI, chloroplast precursor, putative, expressed	/	/	/
