# Supplementary material for: Comprehensive Identification of Key Genes Responsible for Leaf Senescence of Rice (Oryza sativa L.) by WGCNA Using Two Independent Aging Datasets
Source: Plants (Basel). 2025 Aug 30;14(17):2704. doi: 10.3390/plants14172704 (PMC12430195; doi:10.3390/plants14172704)
Supplement: Supplementary file 1 [file plants-14-02704-s001.zip › Table S8.pdf]

Table S8. Function annotation of hub genes in the hub network.

Locus name	Gene product name	Gene symbol	Gene functional information	Reference
**Senescence positively correlated genes**				
Os02g0770800	Nitrate reductase gene	*OsNR2*	Nitrogen use efficiency	Gao et al. 2019
Os05g0530400	Spotted leaf-7	*OsSPL7*	Plant growth and balancing reactive oxygen species; biotic and abiotic stress	Hoang et al. 2019
Os11g0126900	NAC transcription factor	*OsNAC10*	Drought tolerance	Jeong et al. 2010
Os12g0106000	Ferritin gene	*OsFER2*	Ferroptotic cell death and defense response	Nguyen et al. 2022
Os01g0128200	Nuclease PA3, putative, expressed	/	/	/
Os01g0272800	Expressed protein	/	/	/
Os01g0627900	Cytochrome P450 72A1, putative, expressed	/	/	/
Os01g0644000	Tat pathway signal sequence family protein, expressed	/	/	/
Os01g0866200	Histone H3, putative, expressed	/	/	/
Os04g0531900	short-chain Dehydrogenase/reductase, putative, expressed	/	/	/
Os04g0556400	Glucosyltransferase, putative, expressed	/	/	/
Os05g0298200	Expressed protein	/	/	/
Os12g0147800	Phytosulfokines precursor gene	/	/	/
**Senescence negatively correlated genes**				

Os03g0131900	Chloroplast signal recognition particle 43; pale-green leaf	*OscpSRP43; PGL3*	Chloroplast development; chlorophyll synthesis; photosynthesis; leaf senescence	Lv et al. 2015; Ye et al. 2018
Os03g0563300	Magnesium-chelatase subunit ChlI	*OsCHLI*	Chlorophyll biosynthesis; photosynthesis	Zhang et al. 2006
Os08g0435900	Delayed yellowing1-1	*DYE1*	Chlorophyll accumulation; leaf senescence	Yamatani et al. 2018
Os06g0348800	Golden2-like transcription factor	*OsGLK1*	Chloroplast development; programmed cell death	Nakamura et al. 2009; Zheng et al. 2022
Os02g0110200	Hydroperoxide lyase gene	*OsHPL3*	Jasmonic acid pathway; spontaneous cell death	Tong et al. 2012; Tu et al. 2020
Os08g0159500	Rice Zinc finger protein	*OsLSD1; OsLOL1*	Programmed cell death; blast fungus resistance; aleurone layer development	Wang et al. 2005; Wu et al. 2014
Os10g0502400	Glutamyl-tRNA reductase	*OsGluTR*	Chlorophyll biosynthesis; photosynthesis	Jiang et al. 2022
Os07g0147500	10 kDa Photosystem II polypeptide	*PsbR1*	Photosynthesis; cold stress	Li et al. 2017
Os01g0279100	Catalytic subunit of magnesium-protoporphyrin IX monomethyl ester cyclase	*OsCRD1*	Chlorophyll biosynthesis; photosynthesis	Sheng et al. 2017; Wang et al. 2017
Os03g0323200	Mg-chelatase H subunit	*OsChlH*	Chlorophyll biosynthesis; photosynthesis	Jung et al. 2003
Os02g0731700	CONSTANS (CO)-like gene	*Ghd2*	Heading stage; drought tolerance; senescence	Liu et al. 2016
Os02g0128200	BZIP transcription factor	/	/	/

Os03g0333400	Photosystem II 11 kD protein, putative, expressed	/	/	/
Os09g0517000	Expressed protein	/	/	/
Os04g0412500	Bifunctional monodehydroascorbate reductase and carbonic anhydrasenectarin-3 precursor, putative, expressed	/	/	/
Os01g0930800	Os1bglu5 - beta-glucosidase homologue, similar to G. max isohydroxyurate hydrolase, expressed	/	/	/
Os08g0470700	Bifunctional monodehydroascorbate reductase and carbonic anhydrasenectarin-3 precursor, putative, expressed	/	/	/
Os01g0773700	Photosystem II reaction center W protein, chloroplast precursor, putative, expressed	/	/	/
Os07g0546000	Isopentenyl diphosphate isomerase	/	/	/
Os04g0423600	Expressed protein	/	/	/
Os07g0141400	23 kDa polypeptide of the oxygen-evolving complex of photosystem II	/	/	/
Os01g0205300	Rho termination factor, N-terminal domain containing protein, expressed	/	/	/
Os02g0647900	Rice aldehyde dehydrogenase	/	/	/
Os01g0600900	Chlorophyll A-B binding protein, putative, expressed	/	/	/
Os10g0494000	Expressed protein	/	/	/
Os12g0550600	Hypothetical protein	/	/	/
Os08g0382400	Peptidyl-prolyl cis-trans isomerase, chloroplast	/	/	/

	precursor, putative, expressed			
Os02g0596000	Rhodanese family protein, putative, expressed	/	/	/
Os01g0720500	Chlorophyll A-B binding protein, putative, expressed	/	/	/
Os03g0323100	Conserved hypothetical protein	/	/	/
Os10g0493600	Alpha-galactosidase precursor, putative, expressed	/	/	/
Os12g0189300	Carboxyvinyl-carboxyphosphonate phosphorylmutase, putative, expressed	/	/	/
Os05g0560000	Expressed protein	/	/	/
Os12g0124000	Forkhead-associated (FHA) domain-containing protein1	/	/	/
Os01g0940700	Glycosyl hydrolases family 17, putative, expressed	/	/	/
Os01g0144100	Thylakoid lumenal protein, putative, expressed	/	/	/
Os05g0408900	Transketolase	/	/	/
Os04g0457500	Gamma-glutamyltranspeptidase 1 precursor, putative, expressed	/	/	/
Os05g0202500	Expressed protein	/	/	/
Os09g0526700	UDP-galactose/glucose epimerase 3	/	/	/
Os07g0539900	Glucan endo-1,3-beta-glucosidase precursor, putative, expressed	/	/	/
Os02g0716500	Δ12-fatty acid desaturase	/	/	/
Os09g0340400	Expressed protein	/	/	/
Os04g0616700	SHR5-receptor-like kinase, putative, expressed	/	/	/

Os06g0129100	Retinol dehydrogenase, putative, expressed	/	/	/
Os07g0691200	D-alanine--D-alanine ligase family, putative, expressed	/	/	/
Os08g0489300	Methyladenine glycosylase, putative, expressed	/	/	/
Os02g0634700	OsSCP8-putative serine carboxypeptidase homologue, expressed	/	/	/
Os04g0493400	Pathogenesis-related protein-3 chitinase gene	/	/	/
Os07g0516900	RNA recognition motif containing protein, putative, expressed	/	/	/
Os09g0471500	OsWAK82-OsWAK receptor-like cytoplasmic kinase OsWAK-RLCK, expressed	/	/	/
Os03g0704100	PAP fibrillin family domain containing protein, expressed	/	/	/
Os03g0736900	Expressed protein	/	/	/
Os11g0202300	Expressed protein	/	/	/
Os11g0625900	Receptor-like protein kinase precursor, putative, expressed	/	/	/
